# *Epichloë bromicola* Enhances Barley Disease Resistance Through Temporally Coordinated Defense Responses

**DOI:** 10.3390/jof12070484

**Published:** 2026-07-01

**Authors:** Yufan Pang, Wenjing Zhi, Zhenjiang Chen, Kamran Malik, Zhengfeng Wang, Xueqin Han, Jie Jin, Hongshan Deng, Chunjie Li

**Affiliations:** 1 College of Pastoral Agriculture Science and Technology, Lanzhou University, Lanzhou 730020, China; pangyf2024@lzu.edu.cn (Y.P.); zhiwj2025@lzu.edu.cn (W.Z.); chenzhenjiang@lzu.edu.cn (Z.C.); malik@lzu.edu.cn (K.M.); 2 State Key Laboratory of Herbage Improvement and Grassland Agroecosystems, Lanzhou University, Lanzhou 730020, China; 3 Engineering Research Center of Grassland Industry, Ministry of Education, Gansu Tech Innovation Center of Western China Grassland Industry, Lanzhou University, Lanzhou 730020, China; 4 Institute of Economic Crops and Malt Barley Research, Gansu Academy of Agricultural Sciences, Lanzhou 730070, China; wangzhf1006@163.com; 5 Institute of Tropical Eco-Agriculture, Yunnan Academy of Agricultural Sciences, Yuanmou County, Chuxiong 651399, China; hanxueqin@yaas.org.cn (X.H.); jinjie@yaas.org.cn (J.J.)

**Keywords:** *Epichloë bromicola*, barley, disease resistance, multi-omics, antioxidant defense, phenylpropanoid biosynthesis

## Abstract

*Epichloë* endophytes enhance plant defense and biotic stress resistance through mutualistic interactions in natural hosts. However, whether these protective effects are retained in annual non-native hosts such as barley (*Hordeum vulgare* L.) remains unclear. An integrated multi-omics approach was used to characterize defense responses in *Epichloë bromicola*-infected barley (LD1) and endophyte-free barley (CK) following pathogen challenge. The results show that LD1 had 20.0% lower disease incidence and 24.1% lower disease index than CK. LD1 also exhibited 21.1% lower malondialdehyde (MDA) content and higher antioxidant enzyme activities, indicating more effective control of oxidative damage and improved redox homeostasis. Integrated transcriptomic and metabolomic analyses revealed distinct defense responses between LD1 and CK. LD1 showed stronger activation of glutathione metabolism and antioxidant pathways than CK after 6 h of endophyte inoculation. This was consistent with more efficient control of early reactive oxygen species (ROS) dynamics. By 48 h, LD1 preferentially enriched phenylpropanoid biosynthesis and mitogen-activated protein kinase (MAPK) signaling, accompanied by increased accumulation of defense-related metabolites and enhanced structural and chemical defenses. Collectively, the study demonstrates that *Epichloë bromicola* enhances disease resistance in barley by reprogramming host defense dynamics from early redox regulation to late structural and chemical reinforcement.

## 1. Introduction

Barley (*Hordeum vulgare* L.) is a globally indispensable cereal crop, yet its production and grain quality are threatened by diverse pathogen infections, leading to substantial economic losses worldwide [1,2,3]. *Epichloë* endophytic fungi have attracted attention for their role in enhancing host fitness [4,5,6]. These endophytes establish stable, lifelong mutualistic associations with their hosts, providing broad-spectrum resistance against pathogens and environmental extremes [5,6,7,8]. While the protective effects of *Epichloë* are well-documented in perennial forage grasses [6,7], it remains largely subtle whether these symbiotic benefits can be effectively established and maintained in annual cereal crops like barley [8,9]. Furthermore, the molecular mechanisms by which the endophyte modulates the host’s immune landscape during early infection remain poorly understood [4,9].

*Epicoccum nigrum* was selected as the challenge organism because *Epicoccum* species have been reported to cause leaf spot diseases in a range of gramineous plants. Recent pathogenicity studies further demonstrated that several *Epicoccum* species are capable of inducing leaf lesions in Italian ryegrass under controlled conditions [10]. Plants respond to pathogen invasion through coordinated regulation of immune signaling, redox homeostasis, primary metabolism, and secondary metabolism [11,12,13]. Among these processes, ROS function not only as antimicrobial molecules but also as pivotal signaling components that link stress perception to downstream defense activation [12,14]. In addition, metabolic pathways such as glutathione metabolism, phenylpropanoid biosynthesis, and MAPK signaling are closely associated with antioxidant defense and resistance formation [12,13,15]. Although several studies have applied transcriptomic or metabolomic approaches to investigate plant immunity, integrated analyses of transcriptional and metabolic regulation in endophyte-mediated defense responses remain limited, particularly in barley [16].

In this study, the “Landa No. 1” barley–*Epichloë bromicola* symbiotic system was used to investigate interactions between the host and fungal pathogens. Integrated transcriptomic and metabolomic analyses were conducted to characterize host physiological and defense responses during pathogen infection. Enhanced barley disease resistance associated with *Epichloë* colonization was accompanied by coordinated regulation of ROS scavenging and phenylpropanoid biosynthesis.

## 2. Materials and Methods

### 2.1. Plant Materials and Experimental Design

Endophyte-infected barley (*Hordeum vulgare* L., cv. Landa 1, LD1) and endophyte-free barley (CK) were used as experimental materials in this study [17]. The pathogen used for inoculation was *Epicoccum nigrum*. Seeds were surface sterilized with absolute ethanol followed by 5% sodium hypochlorite and then sown in a sterilized substrate consisting of nutrient soil and vermiculite (1:1), which had been autoclaved at 121 °C for 20 min. Plants were grown in a greenhouse for 50 days. Subsequently, leaves were inoculated by spraying a spore suspension of *E. nigrum* at a concentration of 1 × 10^5^ spores mL^−1^ containing Tween 20. After inoculation, plants were covered with plastic bags to maintain high humidity. For all analyses, samples were collected at 0, 6, 24, and 48 h after pathogen inoculation. Three independent biological replicates were included for each treatment at each sampling time point. The same biological replicates were used for physiological measurements, transcriptomic sequencing, and metabolomic analyses.

### 2.2. Determination of Physiological and Biochemical Parameters

Leaf samples were collected at specific time points after pathogen inoculation for physiological measurements. Malondialdehyde (MDA) content was determined using the thiobarbituric acid (TBA) method with slight modifications [18]. Approximately 0.1 g of leaf tissue was homogenized in 1 mL of 0.1% (*w*/*v*) trichloroacetic acid (TCA) and centrifuged at 16,000× *g* for 10 min at 4 °C. The supernatant was mixed with an equal volume of 0.5% TBA (prepared in 20% TCA) and incubated in a boiling water bath (95 °C) for 30 min. The reaction was terminated rapidly in an ice bath, followed by centrifugation. Absorbance was measured at 532 nm and 600 nm, and nonspecific absorbance was subtracted. MDA content was calculated using the extinction coefficient and expressed as nmol∙g^−1^ fresh weight (FW). Hydrogen peroxide (H_2_O_2_) content was measured using the potassium iodide (KI) colorimetric method [19]. Approximately 0.1 g of leaf tissue was homogenized in 0.1% TCA and centrifuged at 16,000× *g* for 10 min at 4 °C. The supernatant was mixed with potassium phosphate buffer (pH 7.0) and KI solution, incubated in the dark for 1 h, and the absorbance was measured at 390 nm. H_2_O_2_ content was calculated based on a standard curve and expressed as µmol g^−1^ FW. Antioxidant enzyme activities were determined following previously described methods with minor modifications [20,21]. Approximately 0.5 g of leaf tissue was homogenized in phosphate buffer under ice-cold conditions and centrifuged at 17,500× *g* for 15 min at 4 °C. The supernatant was used for enzyme activity assays. Catalase (CAT) activity was determined by monitoring the decomposition of H_2_O_2_ at 240 nm. Peroxidase (POD) activity was determined based on the increase in absorbance at 470 nm due to guaiacol oxidation. Superoxide dismutase (SOD) activity was determined using the nitroblue tetrazolium (NBT) photoreduction inhibition method at 560 nm. Enzyme activities were expressed as U·mg^−1^ protein·min^−1^ or U·mg^−1^ protein. Differences between treatments and time points were analyzed using two-way ANOVA followed by Tukey’s HSD test.

### 2.3. Transcriptomic Analysis

Total RNA quality was assessed using an Agilent 2100 Bioanalyzer (Agilent Technologies, Santa Clara, CA, USA). mRNA was subsequently enriched using Oligo(dT) magnetic beads. The enriched mRNA was randomly fragmented by divalent cations, followed by synthesis of double-stranded cDNA using the M-MuLV reverse transcriptase system. The cDNA fragments were then subjected to end repair, A-tailing, and adapter ligation. Fragments of 370–420 bp were selected for PCR amplification. After library quality assessment, sequencing was performed on an Illumina NovaSeq 6000 platform (Illumina Inc., San Diego, CA, USA) using the paired-end 150 bp (PE150) mode. Raw reads were processed by quality control to obtain clean data. Clean reads were aligned to the reference genome using HISAT2 (v2.0.5) [22]. Gene expression levels were calculated using feature Counts (v1.5.0-p3) [23] and normalized as fragments per kilobase of transcript per million mapped reads (FPKM). Differential expression analysis was performed using DESeq2 (v1.42.0) [24]. Genes with adjusted *p*-values (padj) ≤ 0.05 and |log_2_Fold Change| ≥ 1 were considered significantly differentially expressed.

### 2.4. Untargeted Metabolomic Analysis

Approximately 100 mg of tissue samples were extracted with 80% methanol. After centrifugation at 15,000× *g* for 10 min at 4 °C, the supernatant was diluted to a final methanol concentration of 53% for LC–MS analysis. Chromatographic separation was performed using a Vanquish UHPLC system (Thermo Fisher Scientific, Waltham, MA, USA) equipped with a Hypersil Gold column (Thermo Fisher Scientific, Waltham, MA, USA) (1.9 µm, 2.1 × 100 mm). Mass spectrometric data were acquired using a Q Exactive™ HF-X mass spectrometer (Thermo Fisher Scientific, Bremen, Germany) in both positive and negative ion modes. Raw data were processed using Compound Discoverer (v3.3, Thermo Fisher Scientific, Waltham, MA, USA) for peak detection and metabolite annotation. Quality control was conducted using QC samples (coefficient of variation, CV < 30%) and blank samples to ensure data reliability. Multivariate statistical analyses, including principal component analysis (PCA) and partial least squares discriminant analysis (PLS-DA), were performed using R (v4.4.2; R Foundation for Statistical Computing, Vienna, Austria). The robustness of the PLS-DA model was evaluated using permutation tests to avoid overfitting. Differentially accumulated metabolites were identified based on VIP > 1, fold change (FC ≥ 2 or ≤0.5), and adjusted *p*-values (FDR < 0.05) using the Benjamini–Hochberg method. The robustness of the PLS-DA models was evaluated using 200 permutation tests, and model performance was assessed using R2Y and Q2 statistics. Finally, pathway enrichment analysis was conducted using the Kyoto Encyclopedia of Genes and Genomes (KEGG, https://www.genome.jp/kegg/, accessed on 20 June 2026) and the Human Metabolome Database (HMDB, https://hmdb.ca/, accessed on 20 June 2026) [25].

## 3. Results

### 3.1. Physiological Responses of Endophyte-Infected and Endophyte-Free Barley Under Pathogen Infection

**Figure 1:**
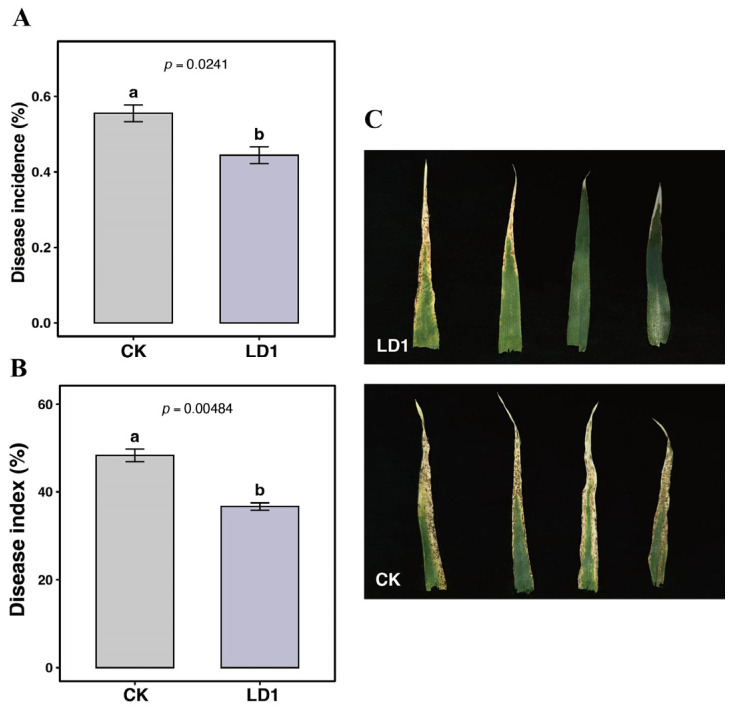
Phylogenetic identification of the pathogen and evaluation of disease resistance in barley. (**A**) Disease incidence (%) of LD1 and CK following pathogen inoculation. (**B**) Disease index of LD1 and CK after infection. (**C**) Representative disease symptoms on barley leaves of LD1 and CK after pathogen inoculation. Data in (**B**,**C**) are presented as mean ± SE (*n* = 3). Different letters indicate statistically significant differences between LD1 and CK according to Tukey’s HSD test (*p* < 0.05). LD1, endophyte-infected barley; CK, endophyte-free barley.

Compared with CK, LD1 exhibited reduced disease development, with disease incidence decreasing from 55.6% to 44.4% and disease index decreasing from 48.3% to 36.7% (Figure 1A,B).

**Figure 2:**
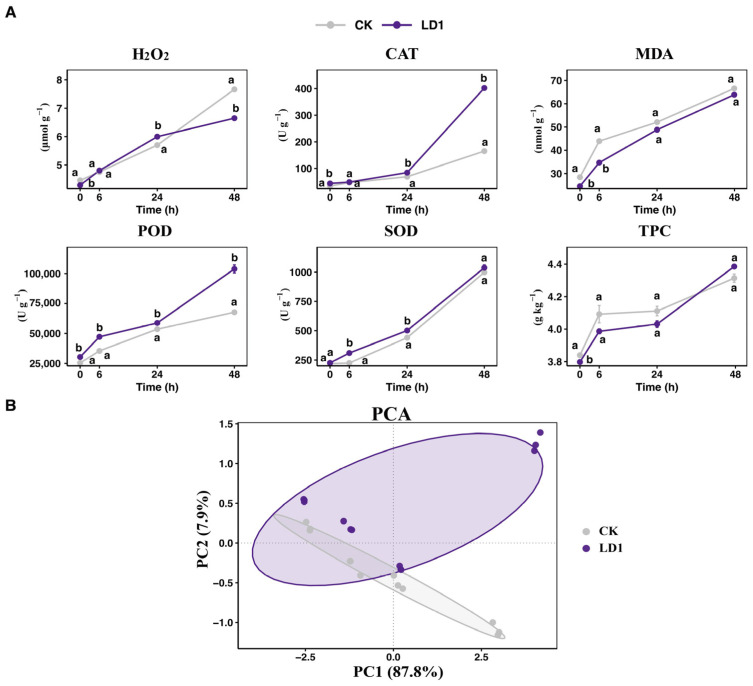
Physiological and biochemical responses of barley to pathogen infection. (**A**) Temporal changes in hydrogen peroxide (H_2_O_2_) content, catalase (CAT) activity, malondialdehyde (MDA) content, peroxidase (POD) activity, superoxide dismutase (SOD) activity, and total phenol content (TPC) in LD1 and CK at 0, 6, 24, and 48 h post-inoculation. (**B**) Principal component analysis (PCA) of physiological traits across all samples. Data are presented as mean ± SE (*n* = 3). Different lowercase letters indicate significant differences between treatments at the same time point (*p* < 0.05). LD1, *Epichloë bromicola*-infected barley; CK, endophyte-free barley.

During pathogen infection, both hydrogen peroxide (H_2_O_2_) and malondialdehyde (MDA) contents increased in LD1 and CK, whereas their temporal accumulation patterns differed between treatments (Figure 2A). At 6 h post-inoculation, MDA content in LD1 was 21.1% lower than in CK, while superoxide dismutase (SOD) and peroxidase (POD) activities were 38.0% and 33.2% higher, respectively. At 24 h, H_2_O_2_ content in LD1 was 5.2% higher than in CK, whereas catalase (CAT), SOD, and POD activities were 22.3%, 13.8%, and 9.7% higher, respectively. By 48 h, LD1 showed 13.2% lower H_2_O_2_ levels together with 143.6% higher CAT activity and 53.4% higher POD activity than CK. Total phenol content (TPC) tended to be higher in LD1 at 48 h, although the difference was not statistically significant. Principal component analysis showed clear separation between LD1 and CK based on physiological traits (Figure 2B).

### 3.2. Metabolomic Analysis Reveals Time-Dependent Metabolic Reprogramming During Pathogen Infection in Barley

**Figure 3:**
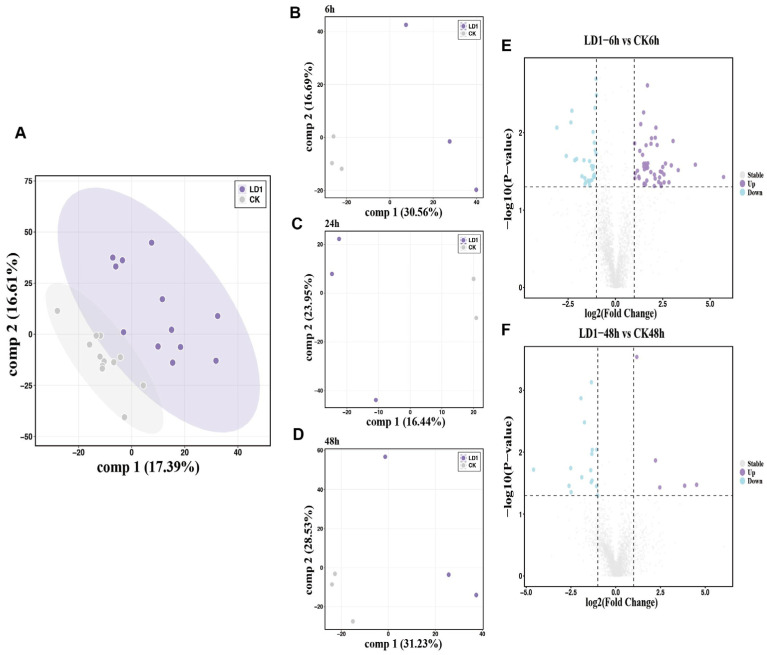
Metabolomic profiling of barley in response to pathogen infection. (**A**) Global PLS-DA score plot showing the overall metabolic differences between LD1 and CK samples. (**B**–**D**) PLS-DA score plots at 6 h (**B**), 24 h (**C**), and 48 h (**D**) post-inoculation. (**E**,**F**) Volcano plots showing differentially accumulated metabolites between LD1 and CK at 6 h (**E**) and 48 h (**F**). Red and blue dots represent significantly upregulated and downregulated metabolites, respectively, while gray dots indicate non-significant metabolites (VIP > 1, *p* < 0.05). LD1, endophyte-infected barley; CK, endophyte-free barley.

Metabolomic profiling showed that endophyte presence altered the metabolic landscape of barley during pathogen infection. Partial least squares discriminant analysis (PLS-DA) revealed clear separation between LD1 and CK in the global metabolic space (Figure 3A). Distinct separation was also observed at each time point (6, 24, and 48 h; Figure 3B–D), with the greatest separation observed at 48 h, indicating progressively increased metabolic divergence over time. Permutation tests confirmed the robustness of the PLS-DA models, as all Q2 intercepts were below zero, indicating that the observed separations were unlikely to result from model overfitting (Figure S1).

Volcano plot analysis further revealed dynamic changes in differentially accumulated metabolites. At 6 h, a total of 76 differential metabolites were identified, including 49 up-regulated and 27 down-regulated metabolites, indicating a predominantly upregulated metabolic response at the early stage. In contrast, at 48 h, only 21 differential metabolites were detected, including 5 upregulated and 16 downregulated metabolites, showing an overall dominance of downregulation and a marked reduction in the number of differential metabolites. Despite the decrease in the number of differential metabolites at 48 h, their distribution became more concentrated, reflecting a shift toward more coordinated and targeted metabolic regulation.

### 3.3. Progressive Amplification of Transcriptional Reprogramming During Infection

**Figure 4:**
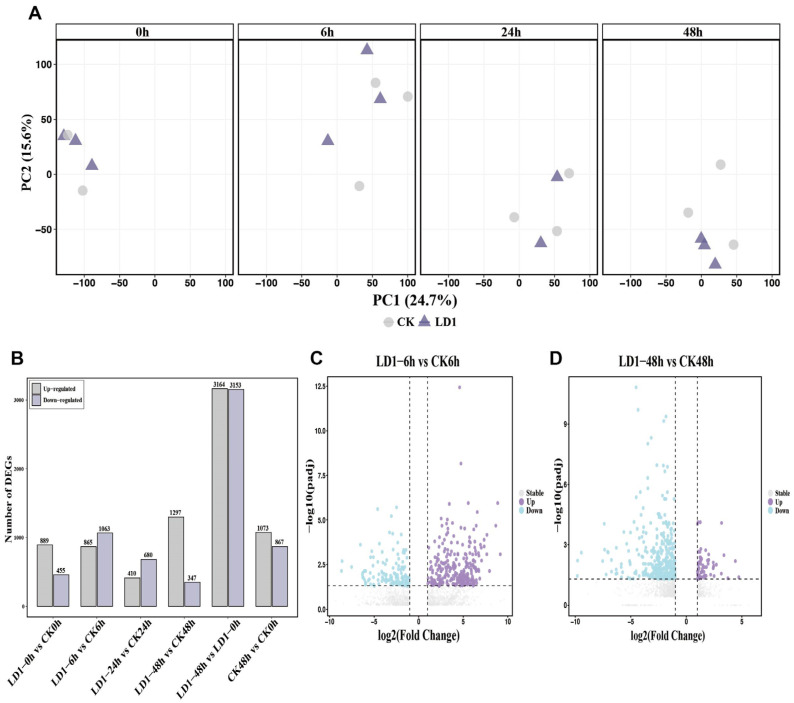
Global transcriptomic responses of barley during pathogen infection. (**A**) Principal component analysis (PCA) showing clear separation of samples across different time points (0, 6, 24, and 48 h). (**B**) Summary of differentially expressed genes (DEGs) between LD1 and CK at each time point. (**C**,**D**) Volcano plots showing the distribution of DEGs at 6 h (**C**) and 48 h (**D**), where red and blue represent upregulated and downregulated genes, respectively. DEGs were identified using thresholds of adjusted *p*-value (padj) ≤ 0.05 and |log_2_(fold change)| ≥ 1. LD1, endophyte-infected barley; CK, endophyte-free barley.

Transcriptomic profiling revealed pronounced transcriptional changes in barley following pathogen infection. Principal component analysis (PCA) showed clear separation of samples across different time points (Figure 4A). At 6 h post-inoculation, 859 genes were upregulated in LD1 compared with CK (DEGs defined as padj ≤ 0.05 and |log_2_Fold Change| ≥ 1), indicating rapid activation of transcriptional responses at the early stage. As infection progressed, the number of differentially expressed genes (DEGs) increased, reaching a maximum at 48 h, with 1257 upregulated genes.

Volcano plots further showed dynamic transcriptional changes at different stages. At 6 h (Figure 4C), DEGs were relatively concentrated along the log_2_(fold change) axis, indicating that most genes exhibited moderate expression changes. In contrast, DEGs at 48 h (Figure 4D) were distributed more broadly along both the log_2_(fold change) and −log_10_(padj) axes, with a greater number of genes showing larger fold changes and lower padj values.

### 3.4. KEGG Enrichment Analysis Reveals Stage-Specific Functional Responses During Pathogen Infection in Barley

**Figure 5:**
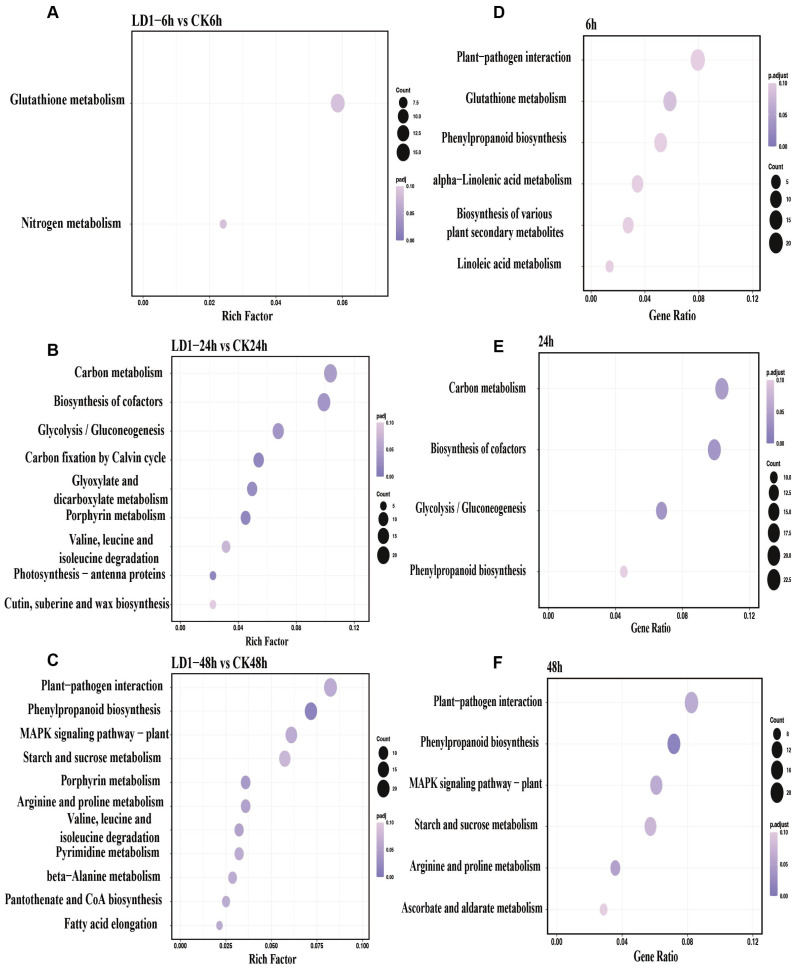
Time-resolved KEGG enrichment analysis of transcriptomic and metabolomic responses in barley. (**A**–**C**) KEGG enrichment of differentially accumulated metabolites between LD1 and CK at 6 h (**A**), 24 h (**B**), and 48 h (**C**) post-inoculation. (**D**–**F**) KEGG enrichment of differentially expressed genes between LD1 and CK at 6 h (**D**), 24 h (**E**), and 48 h (**F**) post-inoculation. The x-axis indicates the enrichment factor (Rich Factor for metabolomic analysis in (**A**–**C**) and Gene Ratio for transcriptomic analysis in (**D**–**F**)), and bubble size represents the number of enriched features. Color gradients indicate adjusted p-values (padj). LD1, endophyte-infected barley; CK, endophyte-free barley.

Kyoto Encyclopedia of Genes and Genomes (KEGG) enrichment analysis further characterized the functional responses of barley at different infection stages (Figure 5). KEGG is a comprehensive database resource for understanding high-level biological functions and pathways (https://www.genome.jp/kegg/). Distinct differences in enriched pathways were observed across stages. At 6 h, both differentially expressed genes and metabolites were mainly enriched in glutathione metabolism and plant–pathogen interaction pathways. At 24 h, enriched pathways were primarily associated with carbon metabolism, glycolysis/gluconeogenesis, and biosynthesis of cofactors. At 48 h, phenylpropanoid biosynthesis and MAPK signaling pathways were significantly enriched, accompanied by increased enrichment of pathways related to secondary metabolism and signal transduction.

### 3.5. Integrated Multi-Omics Analysis Reveals Stage-Dependent Coordination Between Gene Expression and Metabolite Accumulation During Barley Defense Responses

**Figure 6:**
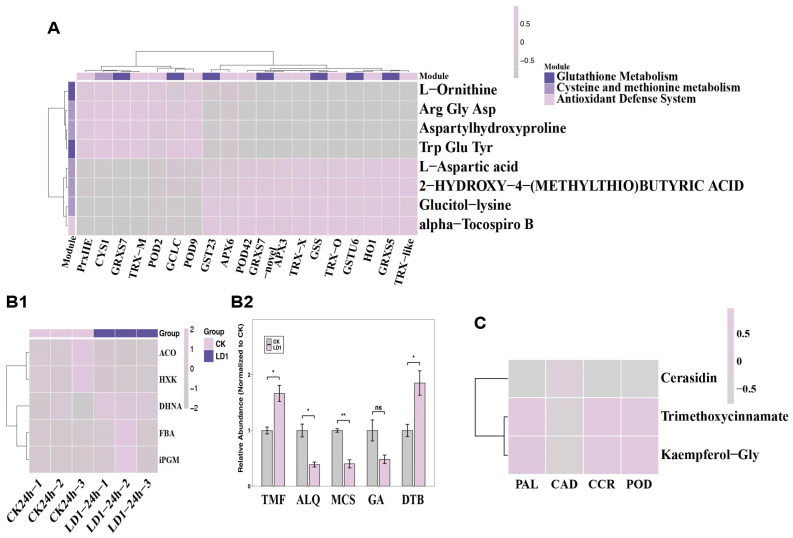
Time-resolved integrative analysis of transcriptomic and metabolomic responses in barley. (**A**) Heatmap showing coordinated changes of genes and metabolites associated with ROS-related pathways at 6 h (early stage), including glutathione metabolism, cysteine and methionine metabolism, and antioxidant defense. Representative metabolites such as L-ornithine, L-aspartic acid, and γ-aminobutyric acid are shown. (**B1**) Integrated analysis at 24 h (middle stage), showing expression patterns of carbon metabolism-related genes.(**B2**) Integrated analysis at 24 h (middle stage), relative accumulation of flavonoid metabolites. (**C**) Heatmap showing coordinated regulation of genes (PAL, CAD, CCR, POD) and metabolites (e.g., cerasidin, trihydroxycinnamate, kaempferol derivatives) involved in phenylpropanoid biosynthesis at 48 h (later stage). Color scales indicate relative expression levels or metabolite abundance. The significance levels are * *p* < 0.05, and ** *p* < 0.01; ns, not significant. LD1, endophyte-infected barley; CK, endophyte-free barley.

To systematically investigate coordinated regulation between the transcriptome and metabolome, key pathways at different infection stages were analyzed integratively (Figure 6). At 6 h (Figure 6A), genes and metabolites associated with ROS-related pathways were jointly analyzed based on pathways commonly enriched in transcriptomic and metabolomic datasets. In the glutathione metabolism, cysteine and methionine metabolism, and antioxidant defense system modules, genes such as GST and TRX showed expression patterns consistent with antioxidant-related metabolites. Early activation of the glutathione system and ROS-related pathways was observed at this stage.

At 24 h (Figure 6(B1,B2)), genes involved in carbon metabolism were significantly enriched and generally upregulated (Figure 6(B1)), while flavonoids and other secondary metabolites accumulated markedly (Figure 6(B2)). Enhanced carbon metabolism and accumulation of defense-related metabolites were observed during this stage. At 48 h (Figure 6C), key structural genes involved in phenylpropanoid biosynthesis, including PAL, CAD, and CCR, were significantly upregulated. Corresponding flavonoids and phenolic acids also accumulated concurrently, with coordinated patterns observed between genes and metabolites during the later stage of defense.

## 4. Discussion

### 4.1. Early-Stage Redox Priming Defines Rapid Defense Onset

Early plant responses to pathogen invasion are typically characterized by rapid ROS accumulation, which plays a dual role as a defense signal and a potential source of oxidative damage [26,27,28,29]. Therefore, precise regulation of ROS homeostasis is crucial for balancing signaling and cellular integrity [26,27,30]. Recent studies suggested that the dynamics of ROS production and scavenging, rather than absolute ROS levels, are key determinants of resistance outcomes [28,31].

At 6 h post-inoculation, LD1 plants exhibited significantly lower accumulation of H_2_O_2_ and MDA than CK, accompanied by enhanced antioxidant enzyme activities. This indicates that endophytes improve the efficiency of redox regulation rather than simply suppressing ROS. Such observations are consistent with the concept of ROS wave-mediated systemic signaling [32,33]. Multi-omics analysis further revealed that glutathione metabolism and associated redox pathways were rapidly activated, with coordinated upregulation of genes such as GST and TRX alongside antioxidant metabolites.

This “early activation” pattern aligns with the defense priming theory, whereby plants acquire enhanced responsiveness prior to severe stress [34,35,36]. In the context of plant–microbiome interactions, endophytes may function as regulators of host immune readiness, enabling faster and more controlled defense activation [35,37,38,39]. Therefore, the present results suggest that enhanced resistance in LD1 was associated with more efficient regulation of early redox dynamics.

### 4.2. Middle-Stage Metabolic Reprogramming Drives Defense Transition

Plant defense responses are energetically demanding and require extensive reconfiguration of metabolic networks to balance growth and defense [40,41,42]. Increasing evidence indicates that primary metabolism actively contributes to immunity rather than serving merely as an energy source [43,44].

At 24 h post-inoculation, pathways related to carbon metabolism, glycolysis, and cofactor biosynthesis were significantly enriched, indicating a major redirection of metabolic flux. This is consistent with the concept of defense-associated metabolic reprogramming [12,43,44,45,46]. In LD1 plants, key genes involved in carbon metabolism were upregulated, suggesting a potential shift in metabolic allocation toward the production of defense-related precursors. Additionally, the accumulation of flavonoids and other secondary metabolites was already evident at this stage, indicating that the intermediate phase represents a critical transition from defense initiation to sustained defense.

### 4.3. Later-Stage Secondary Metabolism Consolidates Defense

At later stages (48 h), plant defense shifts toward the establishment of structural and chemical barriers. Phenylpropanoid metabolism serves as a central hub linking primary metabolism to secondary metabolite production and plays a critical role in plant resistance [44,47,48]. In this study, key structural genes such as PAL, CCR, and CAD were significantly upregulated, accompanied by the accumulation of phenolic acids and flavonoids, demonstrating strong transcription–metabolite coupling [44,45,46].

Meanwhile, MAPK signaling pathways were significantly enriched, suggesting sustained amplification of defense signaling [13,47]. MAPK cascades are known to integrate external stimuli and are frequently associated with defense-related transcriptional responses. Our results indicate that MAPK signaling enrichment coincided with increased phenylpropanoid metabolism and accumulation of defense-related compounds during the later stage of infection.

**Figure 7:**
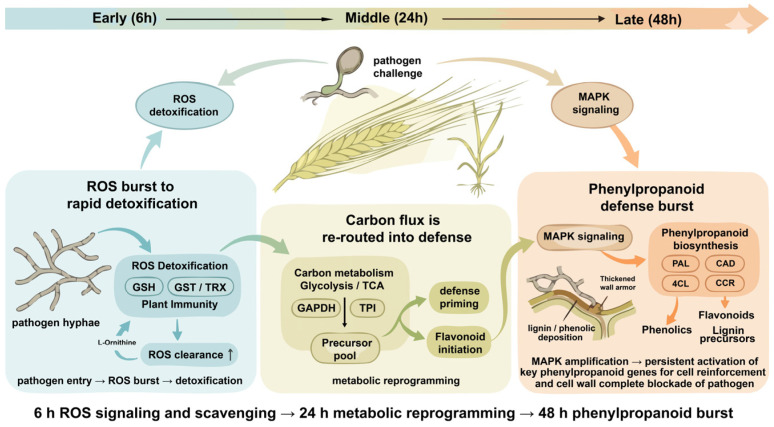
A time-resolved model of endophyte-mediated defense reprogramming in barley. Upon pathogen invasion, barley undergoes a coordinated, stage-specific defense response modulated by endophytes. At the early stage (6 h), a rapid ROS burst is followed by efficient detoxification through glutathione (GSH)-dependent systems and antioxidant enzymes (GST, TRX), maintaining redox homeostasis. At the intermediate stage (24 h), primary metabolism is reprogrammed, with carbon flux redirected into glycolysis and the TCA cycle to generate a precursor pool supporting defense priming and flavonoid initiation. At the later stage (48 h), MAPK signaling was enriched and coincided with increased expression of phenylpropanoid biosynthesis genes, together with the accumulation of phenolic compounds and lignification-related metabolites. Collectively, endophytes enhance disease resistance by orchestrating the temporal coordination of redox regulation, metabolic reprogramming, and secondary metabolism.

This model is consistent with the emerging concept of microbiome-mediated regulation of plant immunity [4,5,38,39,48]. Importantly, the present findings indicate that resistance enhancement is achieved through temporal coordination rather than simply increased defense intensity. Therefore, the present results suggest that endophyte-associated resistance is accompanied by coordinated temporal changes in redox regulation, metabolic reprogramming, and secondary metabolism. (Figure 7).

## 5. Conclusions

This study suggests that endophyte-associated disease resistance is accompanied by temporal changes in host defense responses rather than simply increased defense intensity. Specifically, defense responses followed a coordinated trajectory from early redox regulation to metabolic reprogramming and later reinforcement of structural and chemical barriers. These findings highlight the potential importance of defense timing in plant immunity and provide a conceptual framework for understanding microbiome-associated disease resistance. Future studies should test this proposed defense regulation model in other plant–endophyte systems.

## Data Availability

The raw RNA-seq data generated in this study have been deposited in the Genome Sequence Archive (GSA), National Genomics Data Center (NGDC), China National Center for Bioinformation, under accession number CRA045179. The metabolomics data generated in this study have been deposited in the National Genomics Data Center (NGDC), China National Center for Bioinformation, under accession number OMIX017982. These datasets are publicly available through the corresponding repositories.

## Supplementary Materials

The following supporting information can be downloaded at https://www.mdpi.com/article/10.3390/jof12070484/s1. Figure S1: Permutation tests of PLS-DA models.; Figure S2: Phylogenetic identification of the pathogen isolate used in this study.
