# Supplementary material for: Epichloë bromicola Enhances Barley Disease Resistance Through Temporally Coordinated Defense Responses
Source: J Fungi (Basel). 2026 Jul 1;12(7):484. doi: 10.3390/jof12070484 (PMC13413029; doi:10.3390/jof12070484)

Supplementary Materials

Figure S1. Permutation tests of PLS-DA models.

Permutation-based validation of the global, 6 h, 24 h, and 48 h PLS-DA models. Each panel shows the observed R2Y and Q2 values together with the distributions obtained from 200 label permutations. Q2 values were estimated by leave-one-out cross-validation using the same two-component PLS-DA framework applied in Fig. 3.

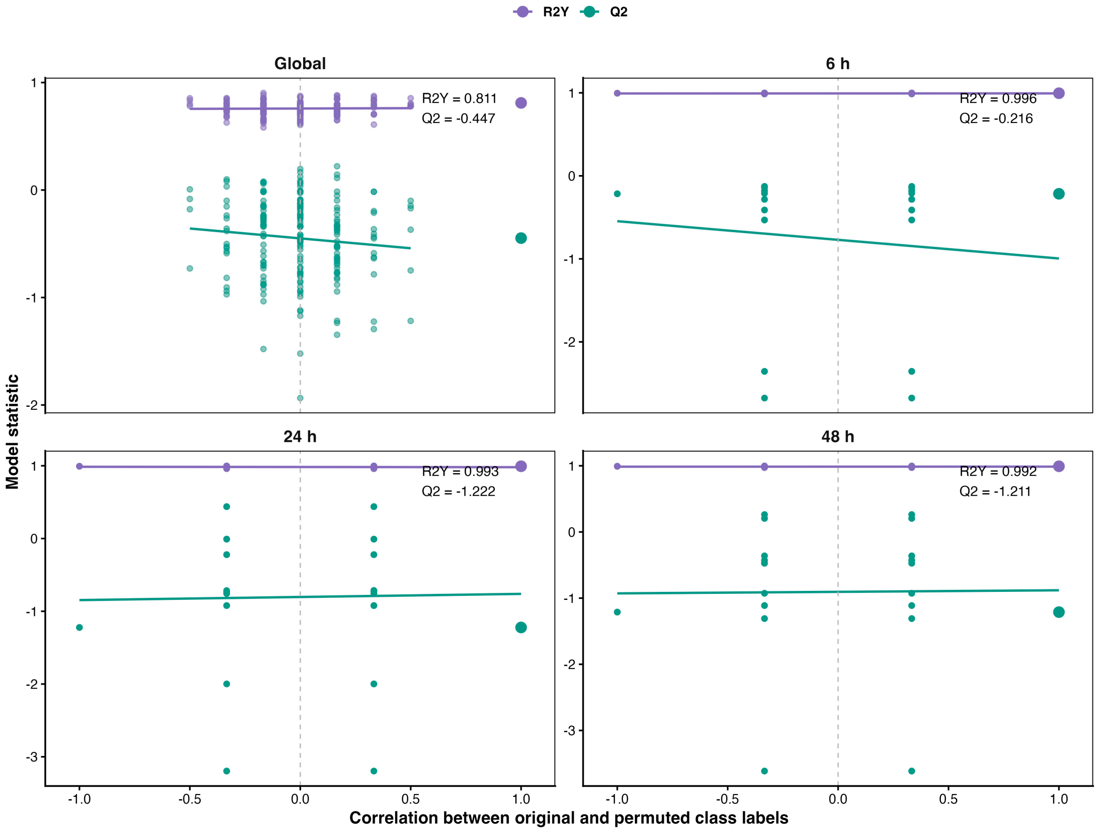

Observed model statistics: Global R2Y/Q2 = 0.811/-0.447; 6 h = 0.996/-0.216; 24 h = 0.993/-1.222; 48 h = 0.992/-1.212. The negative Q2 intercepts obtained from the permutation tests indicate that the observed group separation was unlikely to result from model overfitting.

Figure S2. Phylogenetic identification of the pathogen isolate used in this study.

Phylogenetic tree based on ITS sequences showing the taxonomic position of the pathogen isolate YK1 used in this study. The phylogenetic analysis was performed using the internal transcribed spacer (ITS) region. The isolate YK1 clustered with reference sequences of *Epicoccum nigrum*, supporting its identification as E. nigrum. *Alternaria alternata* was included as the outgroup. Numbers at the nodes indicate bootstrap support values (%).

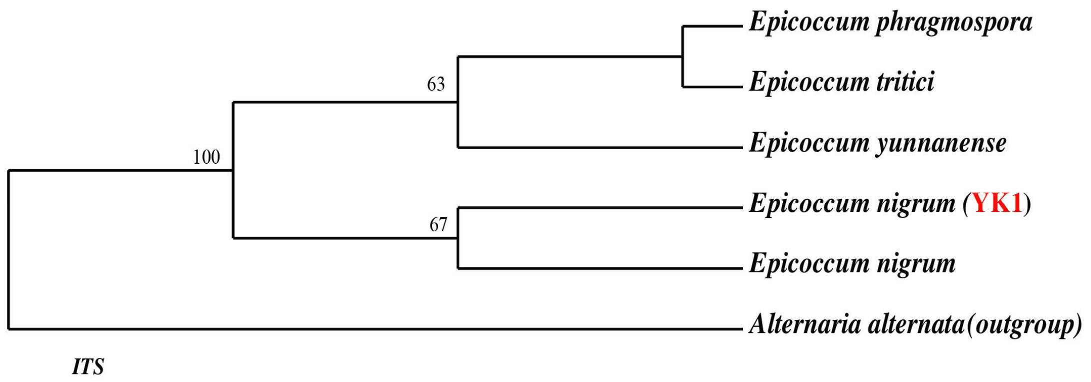

Supplement: Supplementary file 1 [file jof-12-00484-s001.zip › jof-4357548-supplementary.pdf]
